# Early Recovery of Cardiac Function After Hematologic Response in Systemic AL Amyloidosis

**DOI:** 10.1016/j.jaccas.2026.108989

**Published:** 2026-09-23

**Authors:** Aparna Hari, Neeraj Sidharthan, Manoj Unni, Hisham Ahamed

**Affiliations:** a Department of Cardiology, Amrita Institute of Medical Sciences, Kochi, Kerala, India; b Department of Hematology, Amrita Institute of Medical Sciences, Kochi, Kerala, India

**Keywords:** cardiac amyloidosis, echocardiography, functional recovery, global longitudinal strain, left ventricular ejection fraction, light-chain amyloidosis

## Abstract

**Background:**

Cardiac involvement determines prognosis in systemic AL amyloidosis, and functional cardiac recovery typically lags hematologic response by months to years.

**Case Summary:**

A 40-year-old woman with biopsy-proven systemic AL amyloidosis and cardiac, renal, and hepatic involvement received cyclophosphamide, bortezomib, and dexamethasone, achieving rapid deep hematologic response, later consistent with complete response. Left ventricular ejection fraction improved from 40% to 58% and global longitudinal strain (GLS) from −9.2% to −12.1% within 4 months. At 1 year, GLS was −15.8%, and N-terminal pro B–type natriuretic peptide (NT-proBNP) fell from 2,397 pg/mL to 65 pg/mL, with hepatic and renal responses.

**Discussion:**

Cardiac recovery in AL amyloidosis is usually delayed. The rapid improvement in left ventricular ejection fraction, GLS, and NT-proBNP in this case suggests relief from light chain–mediated injury with possible evolving amyloid regression.

**Take-Home Messages:**

Early cardiac functional recovery may accompany deep hematologic response in AL amyloidosis. Serial echocardiography, including GLS, complements NT-proBNP in tracking cardiac response beyond hematologic remission alone.

## Visual Summary

**Figure:**
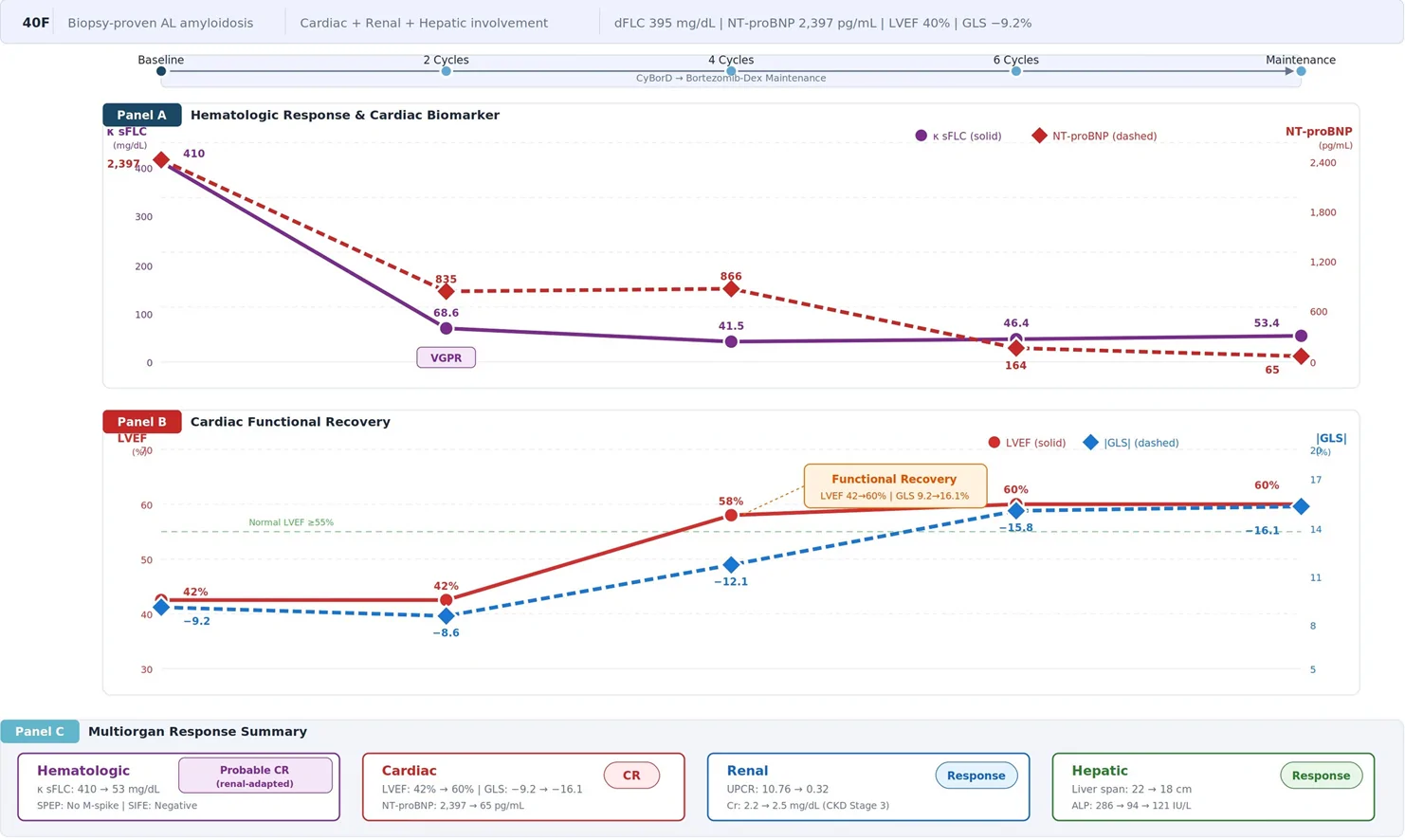

**Visual Summary:**
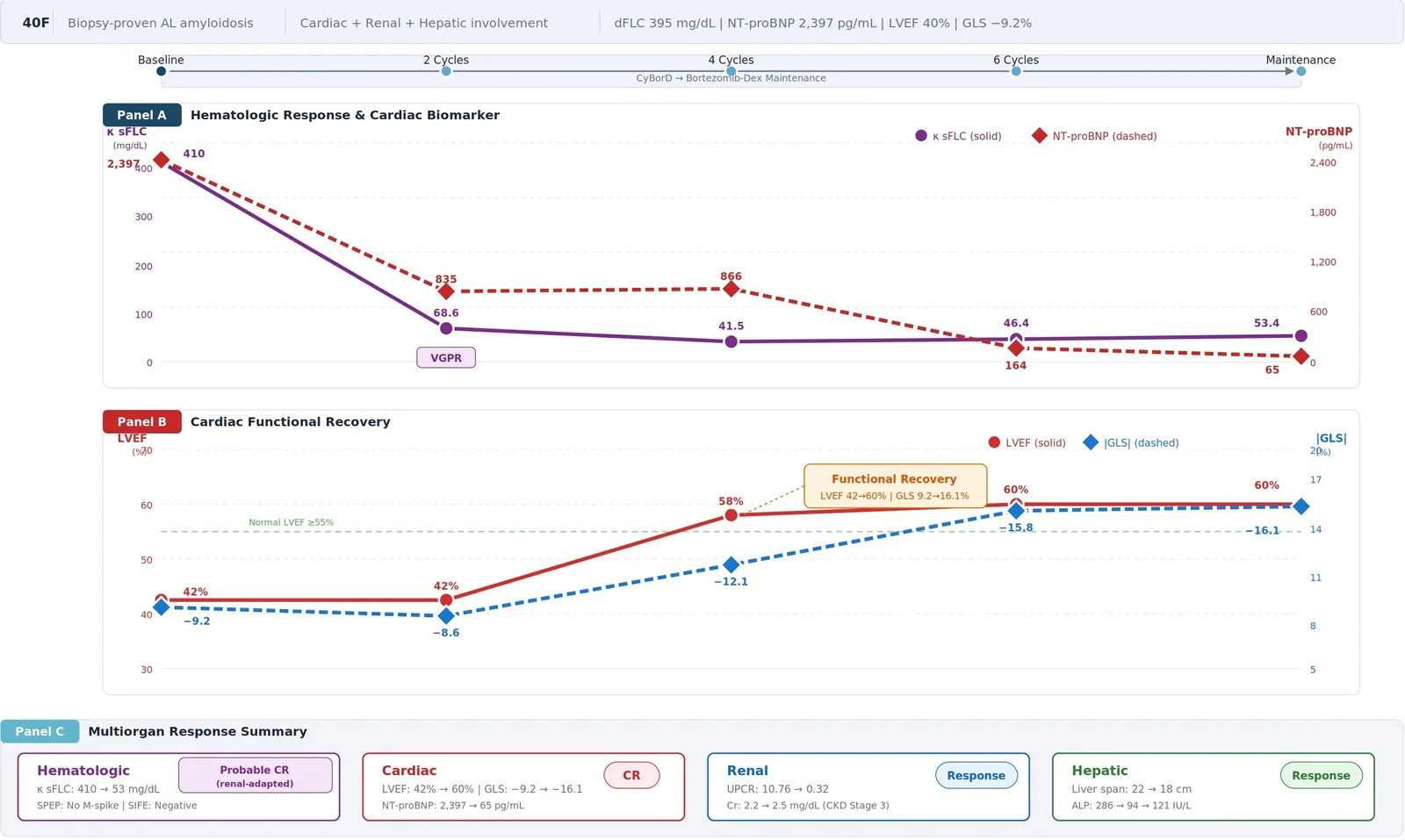
Temporal Kinetics of Multiorgan Response in Systemic AL Amyloidosis Following Initiation of CyBorD Therapy (A) Hematologic response and cardiac biomarker trajectory, both achieving complete response (CR). (B) Serial echocardiographic assessment demonstrating progressive cardiac functional recovery. (C) Summary of multiorgan responses, including sustained hematologic response, cardiac biomarker normalization with echocardiographic recovery, improvement in proteinuria indicating renal response, and reduction in liver span and alkaline phosphatase (AP) consistent with hepatic response. AL = immunoglobulin light chain; CKD = chronic kidney disease; Cr = creatinine; dFLC = difference in involved and uninvolved serum free light chain; GLS = global longitudinal strain; LVEF = left ventricular ejection fraction; NT-proBNP = N-terminal pro–B-type natriuretic peptide; sFLC = serum free light chain; SIFE = Serum immunofixation electrophoresis; SPEP = serum protein electrophoresis; UPCR = urine protein creatinine ratio; VGPR = very good partial response.

Systemic immunoglobulin light chain (AL) amyloidosis is a plasma cell disorder characterized by deposition of misfolded immunoglobulin light chains in multiple organs. Cardiac involvement occurs in approximately 50% to 70% of patients and is the major determinant of prognosis, with historically poor survival when untreated.^1^ Rapid suppression of the amyloidogenic plasma cell clone is therefore essential to halt disease progression and enable organ recovery.

**Take-Home Messages**

• Rapid suppression of amyloidogenic light chains in systemic AL amyloidosis can be associated with early reverse cardiac remodeling and functional recovery.
• Routine longitudinal assessment with natriuretic peptides and echocardiography may help identify early cardiac response beyond hematologic remission alone.

Proteasome inhibitor–based regimens such as cyclophosphamide, bortezomib, and dexamethasone (CyBorD) achieve hematologic responses in a majority of patients, whereas cardiac responses, defined primarily by reductions in N-terminal pro B–type natriuretic peptide (NT-proBNP), occur in a smaller proportion. The addition of daratumumab has further improved hematologic and organ responses.^2^ However, cardiac functional recovery as documented by cardiac imaging remains incompletely characterized. We report a case demonstrating rapid and marked cardiac functional recovery following chemotherapy.

## History of Presentation

A 40-year-old woman presented with 8 months of progressive epigastric discomfort associated with nausea, anorexia, fatigue, and 10-kg unintentional weight loss over 6 months. She also reported frothy urine for several weeks and dyspnea on exertion (NYHA functional class II), without syncope or peripheral edema. Her Eastern Cooperative Oncology Group performance status was 1.

## Past Medical History

Past medical history was notable for hypothyroidism treated with levothyroxine. There was no family history of hematologic disorders or cardiomyopathy.

## Investigations

Examination showed pallor and firm, nontender hepatomegaly extending 8 cm below the right costal margin, without splenomegaly. Blood pressure was 130/90 mm Hg, pulse rate was 80 beats/min, and there was no peripheral edema. Cardiovascular and respiratory examinations were unremarkable.

Abdominal imaging demonstrated marked hepatomegaly (liver span 22 cm on magnetic resonance imaging) with subtle periportal and perivascular branching enhancement in the anterior segments, suggestive of diffuse hepatic involvement. Liver biochemistry was otherwise normal except for elevated alkaline phosphatase (286 IU/L). Evaluation of frothy urine showed nephrotic-range proteinuria (urine protein creatinine ratio [UPCR] 10.76) with serum creatinine of 2.2 mg/dL (estimated glomerular filtration rate 24.7 mL/min/1.73 m^2^). Laboratory studies showed mild microcytic hypochromic anemia (hemoglobin 11.8 g/dL), reactive thrombocytosis, and normal serum calcium (9.56 mg/dL).

**Figure 1:**
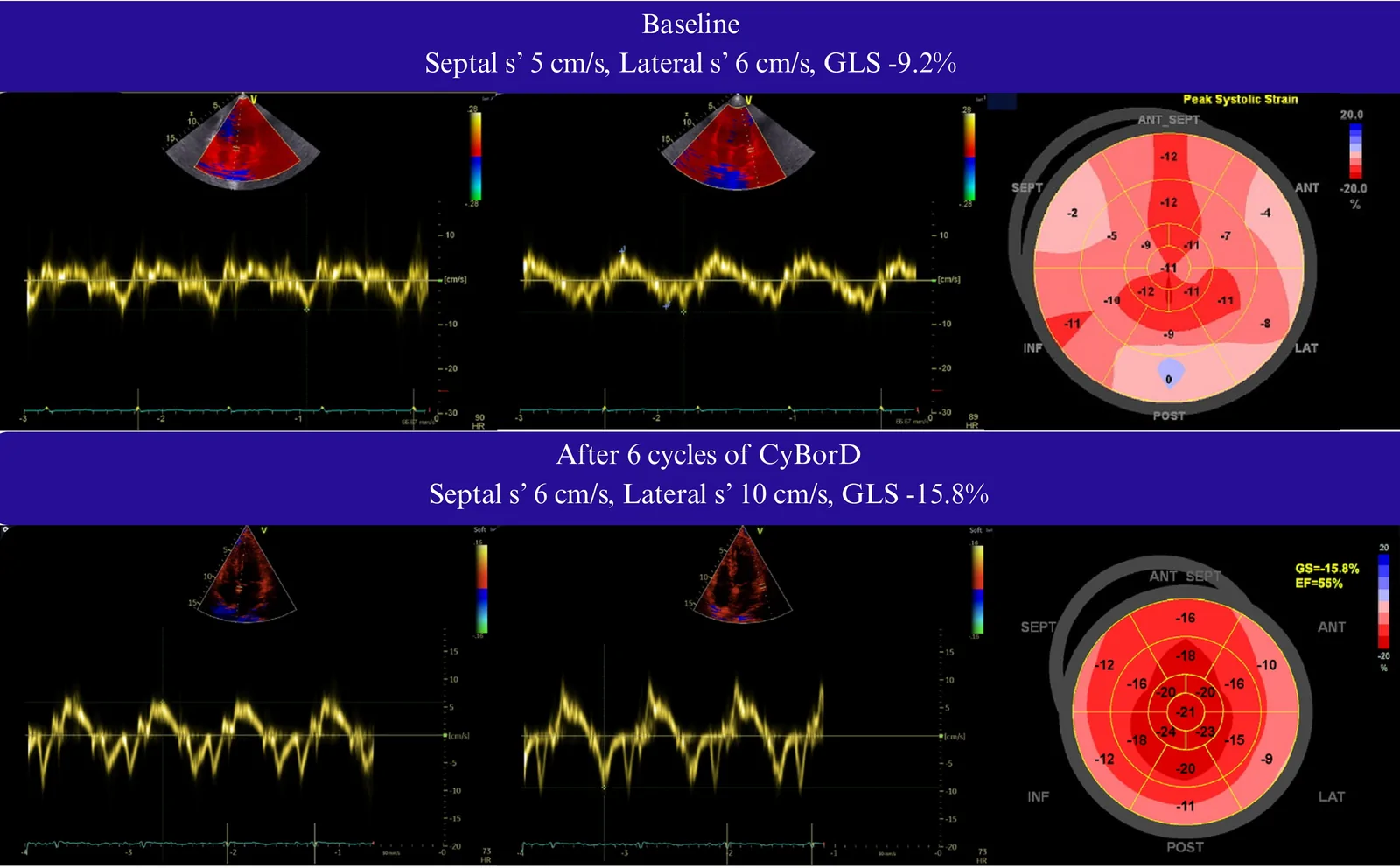
Serial Improvement in Tissue Doppler Velocities and GLS After Chemotherapy for Immunoglobulin Light Chain Cardiac Amyloidosis Baseline echocardiography (top panels) showed impaired longitudinal systolic function with reduced septal and lateral mitral annular tissue Doppler systolic velocities (s′ 5 cm/s and 6 cm/s, respectively) and severely abnormal global longitudinal strain (GLS) (−9.2%). Following 6 cycles of cyclophosphamide, bortezomib, and dexamethasone (CyBorD) (bottom panels), there was interval improvement in annular systolic velocities (septal s′ 6 cm/s, lateral s′ 10 cm/s) and GLS (−15.8%), reflecting early functional myocardial recovery after therapy. CyBorD = cyclophosphamide, bortezomib, and dexamethasone; GLS = global longitudinal strain.

Cardiac biomarkers were elevated (NT-proBNP 2,397 pg/mL; high-sensitivity troponin T 0.035 ng/mL). Electrocardiography showed sinus rhythm with first-degree atrioventricular block and left axis deviation with no prior myocardial infarction pattern. Transthoracic echocardiography demonstrated concentric left ventricular (LV) thickening (septal/posterior wall thickness 1.2/1.6 cm), global LV hypokinesia with left ventricular ejection fraction (LVEF) of 40% to 45%, and severely reduced LV global longitudinal strain (GLS) of −9.2% (Figure 1, Video 1, Video 2, Video 3). Mitral annular tissue velocities were low (septal s′ 5 cm/s; lateral s′ 7 cm/s). Right ventricular systolic function was preserved.

Serum free light chain (FLC) analysis showed markedly elevated κ levels (410 mg/dL) with a difference in involved and uninvolved FLCs of 395 mg/dL. Serum immunofixation demonstrated a faint κ band, whereas serum protein electrophoresis showed no monoclonal spike. Heavy-chain levels were normal.

Liver biopsy showed extracellular eosinophilic material along the sinusoids and portal tracts, consistent with amyloid deposition, with positive Congo red staining. Bone marrow examination revealed 15% plasma cells, including a 3.6% clonal population on flow cytometry, with a normal karyotype.

## Differential Diagnosis

The constellation of hepatomegaly, nephrotic-range proteinuria, LV thickening with systolic dysfunction, and markedly elevated serum FLCs suggested a systemic plasma cell disorder with multiorgan involvement. Key differential diagnoses included systemic AL amyloidosis, multiple myeloma with cast nephropathy, and other infiltrative cardiomyopathies, including transthyretin amyloidosis. The pattern of organ involvement and absence of overt myeloma-defining features favored AL amyloidosis. Hepatic Congo red–positive amyloid deposition together with a clonal plasma cell population in the bone marrow established the diagnosis of systemic light-chain amyloidosis with hepatic, renal, and cardiac involvement.

## Management

Cardiac magnetic resonance (CMR) and mass spectrometry–based proteomic typing of the biopsy specimen were initially planned to assess myocardial involvement and amyloid typing but were deferred because of financial constraints. Given the strong clinical and laboratory evidence for systemic AL amyloidosis with multiorgan involvement and elevated cardiac biomarkers consistent with Revised Mayo 2012 stage III disease, CyBorD was initiated promptly along with supportive therapy, including diuretics.

## Follow-Up

After 2 cycles of CyBorD, the patient achieved a rapid, deep hematologic response, with serum κ decreasing from 410 mg/dL to 68.6 mg/dL. NT-proBNP declined from 2,397 pg/mL to 835 pg/mL, consistent with a cardiac very good partial response. Proteinuria improved (UPCR 10.76-3.01), serum creatinine decreased to 1.8 mg/dL, and echocardiography showed no improvement in LV systolic function.

After 4 cycles, hematologic response was sustained (κ 41.5 mg/dL), with stable NT-proBNP (866 pg/mL). Echocardiography demonstrated marked cardiac functional recovery, with LVEF improving to 58%, LV GLS improving from −9.2% to −12.1%, and mitral annular tissue velocities (s′) improving to 7 cm/s at both the septal and the lateral annulus, consistent with improving longitudinal myocardial function. After 6 cycles, multiorgan response was evident, with persistently suppressed serum FLCs (κ 46.4 mg/dL), reduction in liver span from 22 cm to 18 cm on abdominal ultrasound, and a progressive decline in alkaline phosphatase from 286 IU/L to 167 IU/L, consistent with hepatic response. The LV GLS improved to −15.8%, and LVEF improved to 60% (Figure 1, Video 4, Video 5, Video 6). Although the patient was eligible for consolidation with daratumumab or autologous stem cell transplantation, she opted for maintenance bortezomib and dexamethasone because of financial constraints.

After 18 months of maintenance therapy, serum immunofixation showed no detectable monoclonal protein. Although serum κ remained elevated in the setting of renal impairment, the κ/λ ratio was within the renal reference range, supporting a hematologic complete response. NT-proBNP declined to 65 pg/mL, LVEF remained stable, and proteinuria resolved (UPCR 0.32), consistent with renal response. Serum creatinine stabilized at 2.5 mg/dL, and nephrology follow-up was continued. Serial hematologic, cardiac, renal, and hepatic parameters are summarized in Table 1.

**Table 1:** Serial Hematologic, Cardiac, Renal, and Hepatic Parameters During Chemotherapy

	Baseline	After 2 Cycles	After 4 Cycles	After 6 Cycles	After 18 Months (Maintenance Therapy)
κ sFLC, mg/dL	410	68.6	41.5	46.4	53.4
λ sFLC, mg/dL	15	20.9	11.6	14.9	28.3
NT-proBNP, pg/mL	2,397	835	866	163.5	65
hs-TnT, ng/mL	0.035	—	—	—	—
LVEF, %	40-45	40-45	58	60	60
GLS, %	−9.2	−8.6	−12.1	−15.8	−16.1
UPCR	10.76	3.01	—	0.89	0.32
Creatinine, mg/dL	2.2	1.8	1.53	2.08	2.5
Liver span, cm	22	—	—	18	—
ALP, IU/L	286	241	167	94	121

Values are shown at the specified time points during treatment. Dashes indicate values not measured or not reported at that time point.

ALP = alkaline phosphatase; GLS = global longitudinal strain; hs-TnT = high-sensitivity troponin T; LVEF = left ventricular ejection fraction; NT-proBNP = N-terminal pro–B-type natriuretic peptide; sFLC = serum free light chain; UPCR = urine protein creatinine ratio.

## Discussion

In systemic AL amyloidosis, cardiac involvement is the main determinant of prognosis, and cardiac improvement often lags behind hematologic response.^1^^,^^2^ Although anti–plasma cell therapy suppresses production of amyloidogenic light chains, it does not directly remove existing amyloid deposits, leading to a temporal dissociation between hematologic and organ responses. Cardiac response is therefore defined primarily by biomarkers, particularly a reduction in NT-proBNP of ≥30% and ≥300 ng/L without worsening renal function.^3^ By contrast, imaging evidence of cardiac functional improvement is less well described and usually occurs later, with observational studies reporting a median time to best organ response of 24 to 36 months.^4^

Serial echocardiography, particularly GLS, is a valuable adjunct to natriuretic peptide monitoring. An absolute GLS improvement of ≥2.0% at 1 year has been associated with a 60% reduction in mortality, independent of hematologic response.^5^ Improvements in myocardial work indices have also been associated with better survival. CMR studies suggest that regression of myocardial amyloid burden is infrequent at 1 year and becomes more evident over longer follow-up, primarily in patients with deep hematologic responses.^6^

In our patient, persistent κ elevation in the setting of renal impairment could reflect either residual clonal production or reduced renal clearance, requiring interpretation alongside immunofixation and the renal range κ/λ ratio. In contrast to the usual delayed pattern of organ recovery, she demonstrated rapid cardiac functional recovery within 4 months of chemotherapy initiation, with NT-proBNP decline accompanied by marked improvement in LVEF and GLS. This early and substantial recovery likely reflects rapid suppression of circulating toxic light chains, whereas apparent reduction in LV wall thickness and probable improvement in atrial function suggest that early amyloid regression may also have contributed. The temporal relationship between hematologic response and early cardiac recovery is shown in the Central Illustration.

Several limitations should be acknowledged. CMR was not performed, limiting objective assessment of myocardial amyloid burden. Mass spectrometry–based proteomic typing was also unavailable because of financial constraints, although the clinical profile strongly favored AL amyloidosis. Repeat bone marrow biopsy with flow-cytometric measurable residual disease assessment was not performed during maintenance therapy. This limited definitive distinction between hematologic complete response with persistent polyclonal κ elevation from renal impairment and persistent low-level clonal plasma cell activity. This distinction is clinically relevant because persistent clonality may warrant consolidation or alternative anti–plasma cell therapy despite a favorable clinical trajectory. As a single-case observation, the findings may not be generalizable, and longer follow-up is needed to determine the durability of cardiac recovery.

## Conclusions

This case demonstrates that deep hematologic response in systemic AL amyloidosis may be accompanied by early cardiac functional recovery. Serial assessment with natriuretic peptides and echocardiography, particularly GLS and LVEF, provided objective evidence of myocardial improvement. Although cardiac recovery is usually described later in the disease course, meaningful improvement may occur early in patients achieving deep hematologic response. These findings highlight the importance of early diagnosis and treatment and support incorporation of serial echocardiographic assessment into routine response monitoring.

## Funding Support and Author Disclosures

The authors have reported that they have no relationships relevant to the contents of this paper to disclose.

## Appendix

Video 1 Baseline Parasternal Long-Axis View Baseline transthoracic echocardiography in the parasternal long-axis view demonstrating concentric left ventricular wall thickening with global left ventricular hypokinesia.

Video 2 Baseline Parasternal Short-Axis View Baseline transthoracic echocardiography in the parasternal short-axis view demonstrating concentric left ventricular wall thickening with global left ventricular hypokinesia.

Video 3 Baseline Apical 4-Chamber View Baseline transthoracic echocardiography in the apical 4-chamber view demonstrating concentric left ventricular wall thickening with global left ventricular hypokinesia. Left ventricular ejection fraction was 40% to 45% by the Simpson biplane method.

Video 4 Post-Chemotherapy Parasternal Long-Axis View Transthoracic echocardiography after 6 cycles of chemotherapy in the parasternal long-axis view demonstrating concentric left ventricular wall thickening with normal left ventricular systolic function and apparent reduction in wall thickness compared with baseline.

Video 5 Post-Chemotherapy Parasternal Short-Axis View Transthoracic echocardiography after 6 cycles of chemotherapy in the parasternal short-axis view demonstrating concentric left ventricular wall thickening with normal left ventricular systolic function and apparent reduction in wall thickness compared with baseline.

Video 6 Post-Chemotherapy Apical 4-Chamber View Transthoracic echocardiography after 6 cycles of chemotherapy in the apical 4-chamber view demonstrating normal left ventricular systolic function, with an ejection fraction of 60% by the Simpson biplane method, and apparent reduction in wall thickness compared with baseline.
